# Gray blood late gadolinium enhancement cardiovascular magnetic resonance for improved detection of myocardial scar

**DOI:** 10.1186/s12968-018-0442-2

**Published:** 2018-03-22

**Authors:** Ahmed S. Fahmy, Ulf Neisius, Connie W. Tsao, Sophie Berg, Elizabeth Goddu, Patrick Pierce, Tamer A. Basha, Long Ngo, Warren J. Manning, Reza Nezafat

**Affiliations:** 1 Department of Medicine (Cardiovascular Division), Beth Israel Deaconess Medical Center and Harvard Medical School, 330 Brookline Ave, Boston, MA 02215 USA; 2 Biomedical Engineering Department, School of Engineering, Cairo University, Giza, Egypt; 3 Department of Medicine (Division of General Medicine and Primary Care), Beth Israel Deaconess Medical Center and Harvard Medical School, Boston, MA USA; 4 Department of Radiology, Beth Israel Deaconess Medical Center and Harvard Medical School, Boston, MA USA

**Keywords:** Late gadolinium enhancement, Blood suppression, Myocardial viability, Myocardial infarction

## Abstract

**Background:**

Low scar-to-blood contrast in late gadolinium enhanced (LGE) MRI limits the visualization of scars adjacent to the blood pool. Nulling the blood signal improves scar detection but results in lack of contrast between myocardium and blood, which makes clinical evaluation of LGE images more difficult.

**Methods:**

GB-LGE contrast is achieved through partial suppression of the blood signal using T_2_ magnetization preparation between the inversion pulse and acquisition. The timing parameters of GB-LGE sequence are determined by optimizing a cost-function representing the desired tissue contrast. The proposed 3D GB-LGE sequence was evaluated using phantoms, human subjects (*n* = 45) and a swine model of myocardial infarction (*n* = 5). Two independent readers subjectively evaluated the image quality and ability to identify and localize scarring in GB-LGE compared to black-blood LGE (BB-LGE) (i.e., with complete blood nulling) and conventional (bright-blood) LGE.

**Results:**

GB-LGE contrast was successfully generated in phantoms and all in-vivo scans. The scar-to-blood contrast was improved in GB-LGE compared to conventional LGE in humans (1.1 ± 0.5 vs. 0.6 ± 0.4, *P* < 0.001) and in animals (1.5 ± 0.2 vs. -0.03 ± 0.2). In patients, GB-LGE detected more tissue scarring compared to BB-LGE and conventional LGE. The subjective scores of the GB-LGE ability for localizing LV scar and detecting papillary scar were improved as compared with both BB-LGE (*P* < 0.024) and conventional LGE (*P* < 0.001). In the swine infarction model, GB-LGE scores for the ability to localize LV scar scores were consistently higher than those of both BB-LGE and conventional-LGE.

**Conclusion:**

GB-LGE imaging improves the ability to identify and localize myocardial scarring compared to both BB-LGE and conventional LGE. Further studies are warranted to histologically validate GB-LGE.

**Electronic supplementary material:**

The online version of this article (10.1186/s12968-018-0442-2) contains supplementary material, which is available to authorized users.

## Background

Late gadolinium enhancement (LGE) cardiovascular magnetic resonance (CMR) is an established technique for imaging myocardial fibrosis and left ventricular (LV) scar [1]. In addition, LGE imaging has been shown to have potential for detecting scarred tissues in other cardiac structures, including the left atrium and papillary muscles [2–4]. In LGE, a gadolinium-based contrast agent is injected 10-15 min prior to imaging. The LGE imaging sequence uses an inversion pulse followed by an image acquisition after an inversion delay, at which point the myocardial signal is nulled. The inversion delay is determined prior to the LGE imaging using a Look-Locker sequence [5]. The fast recovery of scar signal (shorter T_1_) lead to scarred tissues appearing bright in LGE images. However, due to the short T_1_ of blood, blood also appears bright and thus leads to low scar-to-blood contrast. This makes detection of myocardial scar in the vicinity of the blood pool challenging [6].

Several approaches have been investigated to improve blood to scar contrast by suppressing the blood pool. One approach utilizes the out-of-slice blood flow to selectively suppress the blood using dual [6] or quadruple [7] inversion pulses. However, sluggish blood flow and the sensitivity of the technique to predetermined timing parameters can be a limitation of this approach [8]. To avoid this limitation, flow-independent approaches have been proposed to null the blood signal based on T_1_ differences among the blood, scar, and normal myocardium. This includes using non-selective dual inversion recovery [8] and blood signal nulling using phase-sensitive LGE [9]. Magnetization preparations before inversion have been also proposed [10–12] for selectively suppressing the blood pool signal relative to the myocardium. We recently proposed a dark-blood LGE (DB-LGE) sequence by inserting the T_2_ magnetization preparation pulse between the inversion pulse and image acquisition [13, 14] to achieve simultaneous nulling of the blood pool and the myocardium. This technique was subsequently used in combination with phase-sensitive inversion recovery (PSIR) [15]. PSIR-based DB-LGE techniques [12, 15–17] are optimized to null the blood pool after the myocardium and thus the negative signal appears darker. A black blood contrast is then generated by means of intensity windowing where blood and myocardium appear black and gray, respectively. This results in a more conspicuous myocardium-blood border due to a high contrast to noise ratio between blood and normal myocardium. A validation study by Francis et al. [16] showed that using T_2_-preparation after the inversion recovery pulse in PSIR LGE increases the observer confidence in detecting subendocardial scar. A flow-independent PSIR-based DB-LGE technique was recently presented and validated by Kim et al. [17]. Magnetization-transfer preparation module was used prior to the inversion recovery pulse to achieve blood suppression. The technique was validated using canine model as a reference standard and human subject data [17].

Prior work of complete suppression of the blood signal [6, 10, 13, 14] can be limited by making the clinical interpretation of LGE images more difficult. For example, simultaneous nulling of healthy myocardium and the blood pool makes it difficult to localize a scar and to assess the transmurality. This approach may also artifactually enhance noise/artifacts in patients without scarring. Therefore, LGE with improved contrast between all 3 tissues (i.e. blood pool, healthy and scarred myocardium) may be preferred to complete blood signal nulling.

In this study, we present a 3D gray-blood LGE (GB-LGE) sequence based on our previous DB-LGE sequence [13, 14] by introducing a parameter optimization approach that allows flexible adjustment of tissue contrast. Numerical simulations, phantoms and in-vivo studies in both humans and a swine model of infarction are used to study the performance of the proposed method.

## Methods

**Fig. 1:**
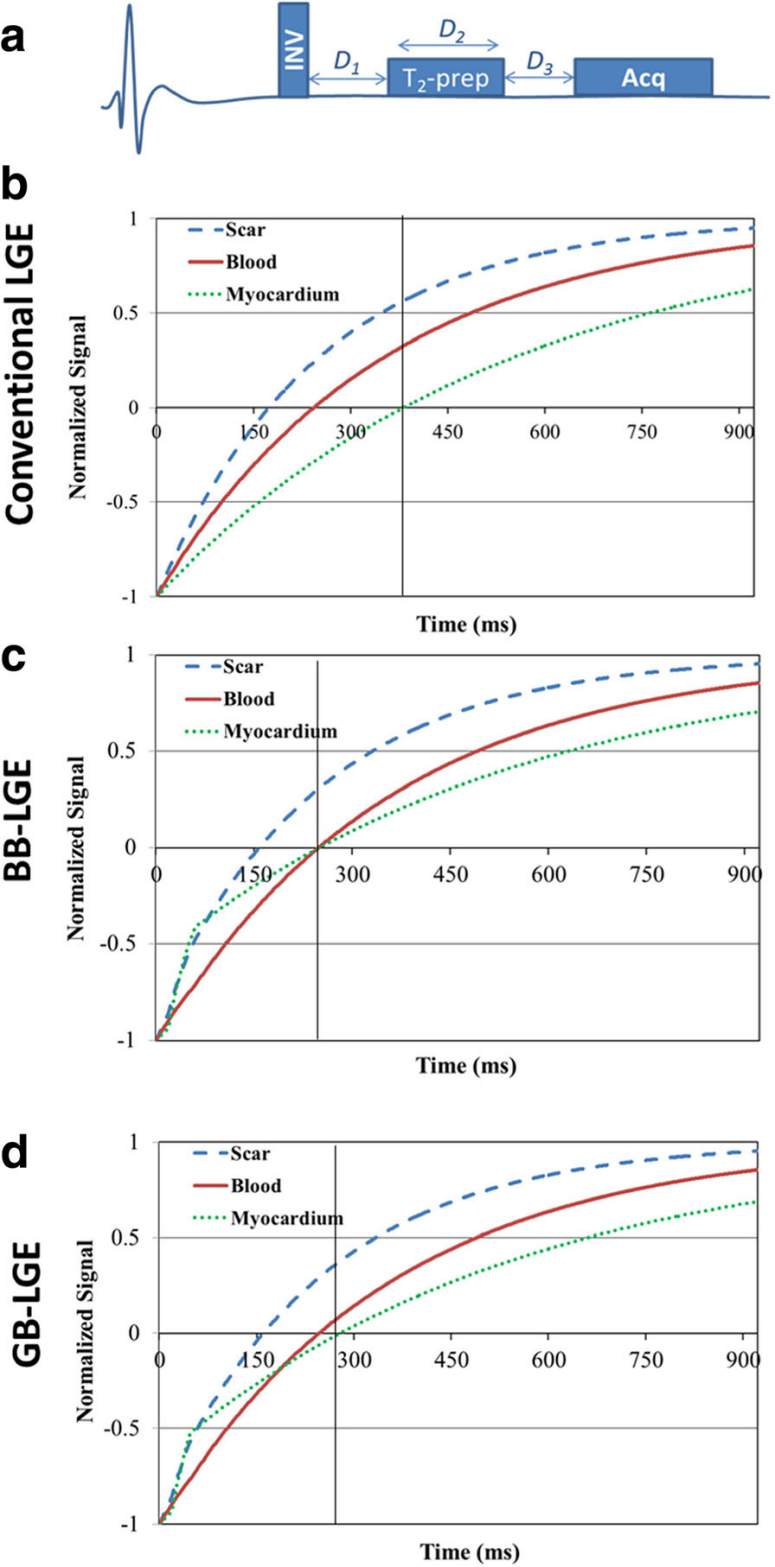
Pulse sequence of the black-blood (BB) late gadolinium enhancement (BB-LGE), gray-blood LGE (GB-LGE) (**a**); and the signal evolution of the different tissues for conventional LGE (**b**), BB-LGE (**c**) and GB-LGE (**d**). All tissue types are assumed having the same (unity) initial signal intensity. Image acquisition time point is indicated by a black vertical line

A block diagram of the DB-LGE sequence is shown in Fig. 1a, indicating the three timing parameters *D*_*1*_, *D*_*2*_ and *D*_*3*_ that are to be determined for GB-LGE [13]. At the beginning of acquisition, the acquired signal is given by [13],

1 $$ {M}^r\left({D}_1,{D}_2,{D}_3\right)=1-{e}^{-\frac{D_3}{T_1^r}}+{e}^{-\frac{D_2}{T_2^r}}{e}^{-\frac{D_3}{T_1^r}}-\left(1+{M}_{ss}^r\right){e}^{-\frac{D_2}{T_2^r}}{e}^{-\frac{\left({D}_1+{D}_3\right)}{T_1^r}}, $$

where, ${T}_1^r$ and ${T}_2^r$ are the T_1_- and T_2_- parameters of the myocardium, blood, or scar, and ${M}_{ss}^r$ is the tissue steady-state magnetization (normalized by the fully-recovered magnetization) available immediately before the inversion-recovery pulse. The subscript *r* can take the symbol: ‘myo’, ‘blood’, or ‘scar’ to indicate the tissue type (myocardium, blood, or scar, respectively). The steady state magnetization, ${M}_{ss}^r$ can be analytically derived given the image acquisition sequence (Appendix).

To allow adjustment of the LGE image contrast, the following cost function, *Q*, is formulated to represent the desired image contrast,

2 $$ Q=\alpha {\left|{M}^{myo}\right|}^2+\left(1-\alpha \right){\left|\beta {M}^{scar}-{M}^{blood}\right|}^2, $$

where, *M*^*r*^ is given by Eq. 1, and *α* and *β* are arbitrary non-negative weights less than 1. The first term in the above equation ensures that the optimal solution minimizes the normal myocardium signal. Minimization of the second term leads to a blood signal that is approximately equal to a desired fraction (*β*) of the scar signal. To obtain a black-blood LGE (BB-LGE) contrast, *β* is set to zero to suppress both the blood and the myocardium signals (Fig. 1c). To partially attenuate the blood signal, *β* is set to a fraction (e.g., 0.1) to obtain GB-LGE contrast (Fig. 1c). In this work, *α* is set to 0.5 in BB-LGE to equally suppress both the blood and the myocardium signals. For GB-LGE contrast, *α* is set to a relatively large value (= 0.9) to prioritize the myocardium nulling over strictly equating the blood signal to a given signal level. The other parameters of the cost function include the T_1_ and T_2_ of the myocardium, the blood, and the scar. The T_1_ values are determined using a T_1_ mapping scan as discussed below, while the T_2_ values of the myocardium, blood, and scar are fixed to previously-reported values equal to 50, 200 and 55 ms, respectively [18, 19]. Changing these fixed values can be shown to have no significant effect on the optimized signal (Additional files 1, 2, 3 and 4).

Since there are only two physical phenomena controlling the optimal solution- namely, T_2_ decay and T_1_ recovery- only two optimization parameters are sufficient to adjust the image contrast. In this work, we use *D*_*2*_ (to control T_2_ decay) and *D*_*3*_ (to control T_1_ recovery), while the parameter *D*_*1*_ is fixed to a predetermined arbitrary value. The optimization problem in Eq. 2 is numerically solved using the Levenberg-Marquadt algorithm [20] implemented in Matlab (Mathworks Inc., Natick, Massachusetts, USA). Numerical iterations were terminated when the changes in *Q* were below 10^− 8^.

### Selecting *D*_*1*_

The feasible range of *D*_*1*_ has a lower limit equal to one-half the duration of the inversion pulse plus the associated crusher gradients. This is determined by the type of inversion pulse; e.g. adiabatic and/or water-selective, and the hardware capabilities of the CMR system. On the other hand, the upper limit of *D*_*1*_ can be determined by observing that the T_2_-preparation pulse needs to start before any of the blood or the myocardium longitudinal magnetizations reaches its nulling time point. Otherwise, it would not be possible to find a time point where both signals are simultaneously nulled. Given that the blood magnetization recovers faster than the myocardium magnetization (due to its shorter T_1_), the upper limit of the feasible range of *D*_*1*_ is given by,

3 $$ {D}_1<{T}_1^{blood}\log \left(1+{M}_{ss}^{blood}\right). $$

A numerical simulation was used to study the effect of fixing *D*_*1*_ on the scar signal. First, 10,000 different combinations of tissues were generated by randomly selecting (using uniform probability distribution) the tissue T_1_ values from different continuous ranges for the blood (${T}_1^{blood}$ = 250-500 ms), myocardium (${T}_1^{myo}$ = 300-600 ms) and scar (${T}_1^{scar}$ = 250-500 ms). To simulate the relative elevated *T*_1_ of the myocardium, caused by rapid washout of contrast from healthy myocardium, only values satisfying ${T}_1^r<{T}_1^{myo}-100$ (with *r* = blood or scar) were used. Other simulation parameters included cardiac cycle duration = 1000 ms, flip angle = 25^o^, TR = 5 ms, number of RF pulses = 24 per cardiac cycle.

For each combination of tissue parameters, *D*_*1*_ was varied from 5 to 100 ms with a step of 5 ms and substituted in Eq. 2 to estimate the optimal *D*_*2*_ and *D*_*3*_ values. Each value of *D*_*1*_ and the corresponding optimal *D*_*2*_ and *D*_*3*_ were then used in Eq. 1 to estimate the scar signal. The mean and standard deviation (mean ± SD) of the scar signal over all tissue combinations were calculated for each *D*_*1*_. The simulation was repeated twice to simulate the scar signal in BB-LGE contrast (*α* = 0.5, *β* = 0) and GB-LGE contrast (*α* = 0.9, *β* = 0.1). The results of this simulation were then used to select an optimal *D*_*1*_ value that yielded the maximum average scar signal.

### Phantom experiments

A phantom of NiCl_2_-doped agarose vials with different T_1_ and T_2_ values was used to demonstrate the impact of different *β* parameters in Eq. 2 as measured by different vial signals. Vials in the phantom (referred to as vial-M, vial-B, and vial-S) contain materials with T_1_/T_2_ parameters mimicking those for the myocardium, blood, and scar (= 598/48, 436/174, and 343/54 ms, respectively). Images were acquired using different imaging parameters based on different *β* values (= 0, 0.01, 0.02, 0.03, 0.04, 0.05, 0.06, 0.07 and 0.08). Imaging with *β*> 0.08 was not feasible because the computed *D*_*2*_ (given the phantom T_1_/T_2_ parameters) was too short. The signal ratios of vial-B and vial-M relative to vial-S were computed.

Imaging was performed on a 1.5 T CMR scanner (Achieva, Philips Healthcare, Best, The Netherlands) with a 32-channel cardiac coil. Electrocardiogram (ECG)-gated three-dimensional (3D) spoiled gradient echo imaging sequence was used with the following parameters: TR/TE = 5.2/2.5 ms, α = 25°, FOV = 320 × 370 × 52 mm^3^, sensitivity encoding rate = 2, acquisition voxel size = 1.5 × 1.5 × 8 mm^3^, acquisition window = 125 ms, low-high phase-encoding order, with 5 startup RF pulses to establish steady-state magnetization, 24 phase encoding line per cardiac cycle, simulated heart rate of 60 bpm. T_1_ values of different vials were measured using modified Look-Locker inversion recovery (MOLLI) 5(1)3 sequence [21] with the following parameters: balanced steady state free precession (bSSFP) acquisition, scan duration = 9 heartbeats, TR/TE = 2.6/1.3 ms, α = 35°, FOV = 300 × 300 × 8 mm^3^, sensitivity encoding rate = 2.0, acquisition voxel size = 2 × 2 × 10 mm^3^, acquisition window = 242 ms, linear phase-encoding order, with 10 startup radiofrequency (RF) pulses to establish steady-state magnetization, and 93 phase encoding lines per cardiac cycle. Equation 2 was then used to estimate the imaging parameters of the 3D GB-LGE and BB-LGE sequences.

### In-vivo experiments

The imaging protocol was approved by our institutional review board and the study was HIPAA-compliant. All subjects provided written informed consent to participate in this study. For the animal study, the research protocol was approved by the Institutional Animal Care and Use Committee and conformed to the Position of the American Heart Association on Research Animal Use as well as to the Declaration of Helsinki.

#### Humans

We prospectively recruited 45 patients (28 males, 53 ± 13 years) referred for CMR evaluation of myocardial viability. In 27 patients (53 ± 14 years, 14 males), each patient was imaged using 3 different viability sequences: conventional 3D LGE, followed in random order by 3D BB-LGE (*β* = 0, *α* = 0.5) and 3D GB-LGE (*β* = 0.1, *α* = 0.9). In a second group of 18 patients (53 ± 13 years, 14 males), only the conventional 3D LGE and the 3D GB-LGE (*β* = 0.1, *α* = 0.9) were used to minimize total scan time. Patients were scanned 15-25 min after bolus infusion of a 0.15 mmol/kg dose of Gadobutrol (Bayer Healthcare, Berlin, Germany).

For all scans, ECG-gated free-breathing 3D spoiled gradient echo based imaging sequence was used to acquire the images using a 32-channel cardiac coil. The imaging parameters for the 3D BB-LGE and 3D GB-LGE techniques were: TR/TE = 5.2/2.5 ms, α = 25°, FOV = 300-400 × 300-400 × 80-120 mm^3^, sensitivity encoding rate = 1.5-2.0, acquisition voxel size = 1.5 × 1.5 × 4-6 mm^3^, acquisition window = 125 ms, low-high phase-encoding order, with 5 startup RF pulses to establish steady-state magnetization, 24 phase encoding line per cardiac cycle, and spectral pre-saturation inversion recovery based fat suppression. Malcom-Levitt (MLEV) composite refocusing pulses were used for T_2_ preparation [22]. Conventional 3D LGE imaging was performed using similar imaging parameters with 18-24 phase encoding lines per cardiac cycle and acquisition window = 95-125 ms. A Look-Locker scouting sequence [5] was used to determine the proper inversion time delay of the LGE sequence. Respiratory motion was compensated using a diaphragmatic 2D pencil beam adaptive navigator gating (window = 7 mm) [23] with slice-tracking. Assuming 100% navigator efficiency, the scan time was 0.8-2.2 min with number of reconstructed (interpolated) slices = 15-42. A factor of 2 oversampling was used in slice orientation.

To estimate the T_1_ values of the myocardium and the blood, T_1_ mapping was performed using a single slice MOLLI sequence 5(1)3 [21] with the following parameters: mid-ventricular short-axis, bSSFP acquisition, scan duration = 9 heartbeats, TR/TE = 2.6/1.3 ms, α = 35°, FOV = 300-400 × 300-400 × 10 mm^3^, sensitivity encoding rate = 2.0, acquisition voxel size = 2 × 2 × 10 mm^3^, acquisition window = 242 ms, linear phase-encoding order, with 10 startup RF pulses, and 93 phase encoding lines per cardiac cycle. The scar T_1_ in Eq. 1 is set equal to 0.7 that of the blood. The T_1_ map for each patient was automatically reconstructed on the scanner main computer and the operator manually selected two regions inside the myocardium and the blood (using the standard graphical interface of the scanner) and the average T_1_ values were used to estimate the optimal imaging parameters. A workstation next to the scanner (a computer with 2.8 GHz Intel Xeon processor and 16 GB RAM) was used to run the optimization algorithm.

#### Animals

Five Yorkshire swine underwent 180 min balloon occlusion of the mid left anterior descending (LAD) coronary artery to create an ischemia-reperfusion mediated myocardial infarction (as previously described [13]). After 8 weeks, CMR scanning was performed. The LGE sequences were performed within 15-35 min after bolus infusion of a 0.2 mmol/kg dose of gadobenate dimeglumine (MultiHance, Bracco, Rome, Italy). Free-breathing 3D BB-LGE and 3D GB-LGE sequences were performed on the animals with the following parameters: TR/TE = 5.4/2.6 ms, α = 25°, FOV = 360-400 × 360-400 × 90-120 mm^3^, sensitivity encoding rate = 1.8, acquisition voxel size = 1.5 × 1.5 × 3 mm^3^, 35 slices, acquisition window = 135 ms, low-high phase-encoding order, with 5 startup RF pulses to establish steady-state magnetization, 24 phase encoding lines per cardiac cycle, and spectral pre-saturation inversion recovery based fat suppression. Acquisition time assuming 100% gating efficiency was 2-2.5 min. The T_1_ values of the different tissues were estimated using a MOLLI sequence as described in the human subjects section. A 3D PSIR LGE imaging [24] was performed and used for comparison with the proposed sequences. The imaging parameters of the 3D PSIR LGE were: TR/TE = 5.6/2.7 ms, α = 15°, FOV = 360-400 × 360-400 × 120 mm^3^, sensitivity encoding rate = 2.3, acquisition voxel size = 1.5 × 1.5 × 10 mm^3^, 12 slices, acquisition window = 115 ms, low-high phase-encoding order, with 10 startup RF pulses to establish steady-state magnetization, 17 phase encoding lines per cardiac cycle, and spectral pre-saturation inversion recovery based fat suppression. Acquisition time assuming 100% gating efficiency was 2.4-2.5 min.

### In-vivo image analysis

The in-vivo datasets (45 human subjects and 5 swine) were quantitatively analyzed to compare the contrast resulting from the three sequences: GB-LGE, BB-LGE, and conventional LGE. Regions of interest were drawn within the myocardium, blood-pool and the hyper-enhanced area in the GB-LGE images and the corresponding location in the BB-LGE and the conventional LGE images. The slice with the maximum scar extent was selected for analysis. Due to the absence of a ground truth (i.e., histology) to verify the hyper-enhancement seen with each technique, only hyper-enhancements that were visible on all three sequences were included in the analysis. Quantitative analysis was performed for the scar-to-blood, scar-to-myocardium, and blood-to-myocardium relative contrast. The relative contrast of tissue A to tissue B is defined as the difference between the average signal of A and B divided by the average signal of B.

Subjective analysis for the dataset was performed by two independent readers (CWT with 10 years CMR experience and UN, a Level-III SCMR-accredited reader). Hyper-enhancements within the LV and the papillary muscles were assessed. For each dataset, readers independently assigned ‘yes’, ‘no’, or ‘uncertain’ to specify whether a scar was present or not. Also, the readers independently evaluated the image quality of each dataset and specified whether it is of acceptable or poor quality. An image with acceptable quality was defined to have proper myocardium nulling and no severe imaging artifact that might limit the diagnosis. The diagnostic value of each technique was subjectively evaluated in humans using two measures: scar detection and scar localization. The former measures the technique’s ability to determine the presence or absence of hyper-enhancements in the LV or papillary muscles and was evaluated using a 4-point scale from 1 = challenging to 4 = easy. A similar scale was used to measure the ability to localize the scarring and determine its pattern and extent. In animals, the models involved only myocardial infarction and thus only the ability of the different techniques to localize the scarred tissues was evaluated. Any disagreement in the presence or absence of scar was reviewed in a subsequent consensus reading by both readers.

### Statistical analysis

For human subject dataset analysis, a non-parametric Friedman statistical test with Bonferroni adjustment was used to test significant differences in the relative scar contrast or qualitative assessment scores among the GB-LGE BB-LGE, and conventional LGE sequences. Additionally, the Wilcoxon signed-rank test with Bonferroni-Holm adjustment was used to perform pairwise comparisons of the different LGE sequences. In these tests, the pairwise test between GB-LGE and conventional LGE images was performed using the two groups of patients (*n* = 45). In the tests involving BB-LGE, data from the first group of patients (*n* = 27) were used to allow paired comparison. Analyses were done using Matlab (Mathworks Inc., Natick, MA). Statistical significance was set at type-I error of 0.05. Due to the small size of the animal dataset (*n* = 5), we did not perform any statistical analysis and only provided the actual subjective assessment and all images.

## Results

**Fig. 2:**
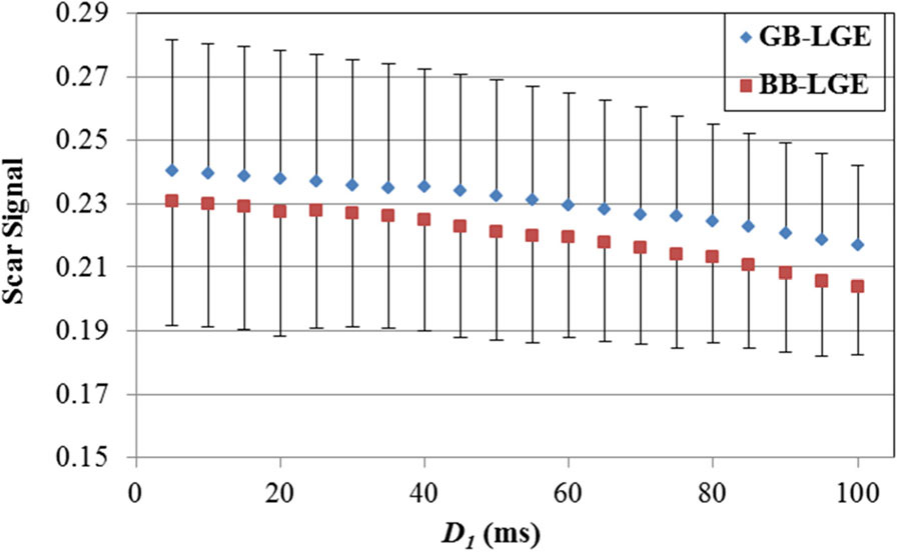
The simulated scar signal resulting from solving the optimization problem at different fixed values of *D*_*1*_ (from 5 ms to 100 ms). Each data point is averaged over 10,000 different combinations of tissue T_1_ and T_2_ values, with error bars representing standard deviation. The figure shows that the lower the value of *D*_*1*_ is, the higher the scar signal. BB = black blood, GB = gray blood, LGE = late gadolinium enhancement

The numerical simulation of the effect of *D*_*1*_ on the resulting scar signal is summarized in Fig. 2. The maximum scar signal (= 0.23 ± 0.04 and 0.24 ± 0.04 in BB-LGE and GB-LGE, respectively) occurred at the lowest value of *D*_*1*_ (= 5 ms), and continued to decrease with increased *D*_*1*_. Therefore, to obtain maximum scar signal, *D*_*1*_ should be set to its minimum feasible value. Based on this finding, *D*_*1*_ was set to the minimum value allowing the use of adiabatic and/or water-selective inversion-recovery pulses in addition to the crushing gradients (= 20 ms).

**Fig. 3:**
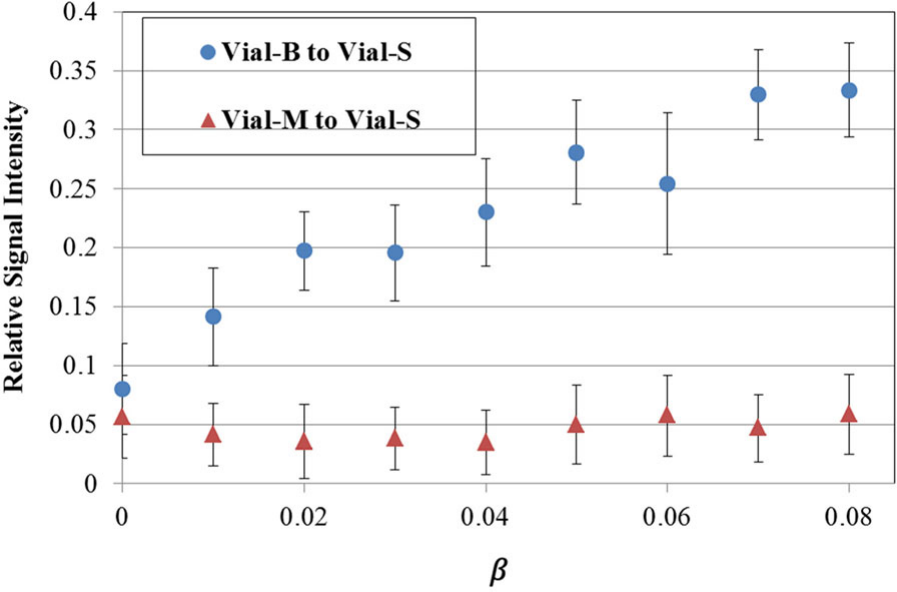
A plot of the vial-B (i.e. blood) to vial-S (i.e. scar) signal ratio, and vial-M (i.e. myocardium) to vial-S signal ratio computed from phantom images acquired with different ***β***. The vial-B to vial-S signal ratio increases with ***β***, while the vial-M to vial-S signal ratio is consistently suppressed for all values of ***β***. Error bars represent standard deviations

The phantom experiment demonstrated flexible adjustment of the signal from different vials (Fig. 3). Increasing *β* from 0 to 0.08 resulted in a gradual increase of the signal ratio between vial-B and vial-S from 0.07 to 0.35. For all values of *β*, the signal ratio of vial-M to vial-S was below 0.06 showing a suppression of the myocardium-mimicking vial.

For the in-vivo imaging, the estimated timing parameters were variable among different patients depending on T_1_ tissue parameters. The estimated parameter *D*_*2*_ for the BB-LGE and GB-LGE sequences varied from 7 to 26 ms (median = 15 ms), and from 4 to 28 ms (median = 9 ms), respectively. The parameter *D*_*3*_ varied from 146 to 208 ms (median = 183 ms), and from 184 to 273 ms (median = 217 ms) for the BB-LGE and GB-LGE sequences, respectively. The computation time for determining the optimal parameters was less than 1 s.

**Fig. 4:**
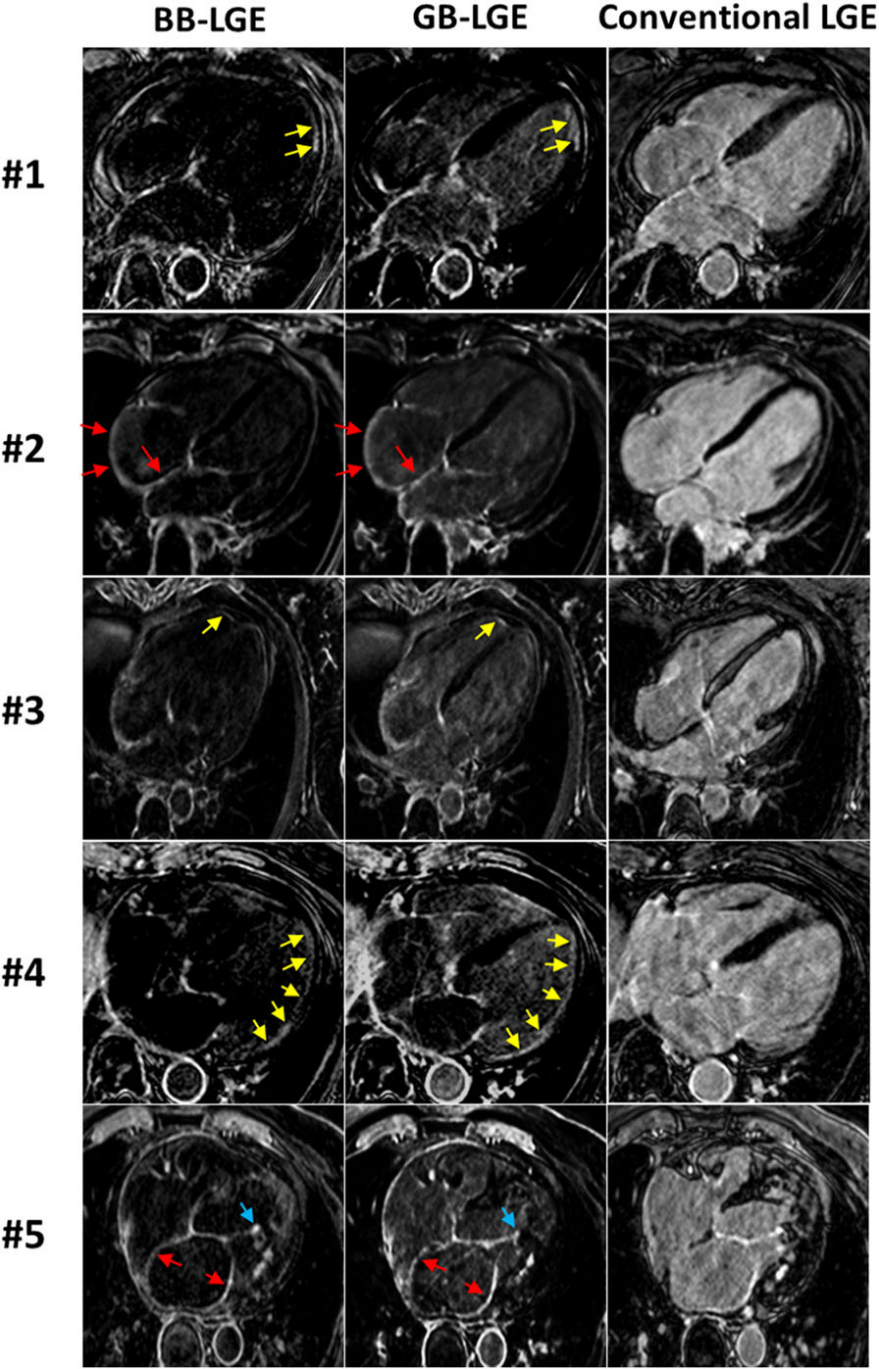
Comparison of the black-blood (BB) late gadolinium enhancement (BB-LGE), gray-blood LGE (GB-LGE), and conventional LGE images in four patients (axial views). Arrows indicates visible hyper-enhancements of atrium (red), sub-endocardial scar (yellow), chordae tendineae and papillary muscles (blue)

Both GB-LGE and BB-LGE showed improved blood and scar contrast (Fig. 4), however GB-LGE images showed improved visualization of healthy myocardium. Compared to the conventional LGE images, suppression of the blood signal in GB-LGE and BB-LGE sequences allowed clear visualization of the hyper-enhanced atria (red arrow), left and right ventricular hyper-enhancement (yellow arrows), and chordae tendineae and papillary muscles (blue arrows) in both GB-LGE and BB-LGE images (Fig. 4 and Additional files 2 and 3).

The Friedman test of the quantitative analysis of the human subjects’ images showed a significant difference among the three sequences in the scar-to-blood relative contrast (*P* < 0.001). Pairwise comparisons indicated that the scar-to-blood relative contrast in GB-LGE (1.1 ± 0.5) was significantly higher than that in the conventional LGE images (0.6 ± 0.4) with *P* < 0.001 (Table 1). Due to the complete nulling of the blood signal, the BB-LGE scar-to-blood relative contrast (3.61 ± 1.83) was significantly higher than that of both GB-LGE (*P* < 0.001) and conventional LGE (*P* < 0.001). The scar-to-myocardium relative contrast in GB-LGE, BB-LGE, and standard LGE were 5.1 ± 3.1, 6.1 ± 4.1, and 5.9 ± 3.5, respectively (Table 1). The scar-to-myocardium relative contrast of the conventional LGE was comparable to that of GB-LGE (*P* = 0.19) and BB-LGE (*P* = 0.59). The GB-LGE showed significantly higher scar-to-myocardium relative contrast than that of BB-LGE (*P* = 0.023) which could be due to the shorter T_2_ preparation pulses in GB-LGE compared to BB-LGE. As expected, suppression of the blood signal has significantly reduced the blood-to-myocardium relative contrast in GB-LGE (1.7 ± 1.4) and BB-LGE (0.7 ± 1.2) compared to conventional LGE (3.8 ± 3.5) with *P* < 0.001 for all pair-wise tests (Table 1).

**Table 1:** Mean ± standard deviation of the slice scar-to-blood and scar-to-myocardium and myocardium-to-blood relative contrast in the black-blood (BB) late gadolinium enhancement (BB-LGE), gray-blood LGE (GB-LGE), and conventional LGE sequences in the human dataset

	Scar-to-Blood relative contrast	Scar-to-Myocardium relative contrast	Blood-to-Myocardium relative contrast
GB-LGE^a^	1.11 ± 0.51	6.12 ± 4.17	1.69 ± 1.45
BB-LGE^a^	3.61 ± 1.83	5.13 ± 3.17	0.73 ± 1.28
Conventional LGE^a^	0.56 ± 0.36	5.96 ± 3.57	3.88 ± 3.53
Friedman Test (*p*-value)	< 0.001	0.028	< 0.001
BB-LGE vs. LGE^b^	< 0.001	0.59	< 0.001
GB-LGE vs. LGE^b^	< 0.001	0.19	0.005
GB-LGE vs. BB-LGE^b^	< 0.001	0.023	< 0.001

^a^Data represent the mean ± standard deviation of the relative contrast

^b^Data represent the p-value of the pairwise Wilcoxon signed-rank test

In the subjective assessment, both readers assigned an image quality score of ‘good’ to 44 GB-LGE datasets, 44 BB-LGE datasets, and 42 conventional LGE datasets. Both readers confirmed presence of LV scar in 17 datasets (out of 45): 9 in patient group 1 and 8 in patient group 2 (Table 2). All cases were correctly identified in GB-LGE and BB-LGE images by both readers. Among these 17 datasets, reader 1 and reader 2 missed the presence of the scar in 5 and 4 cases in the conventional LGE images, respectively. None of the GB-LGE datasets was assigned an ‘uncertain’ score by either of the readers. In contrast, readers 1 and 2 assigned ‘uncertain’ assessment scores to 2 and 4 conventional LGE datasets, respectively and both assigned ‘uncertain’ to 1 BB-LGE dataset. Also, more papillary scar were identified by both readers in GB-LGE compared to BB-LGE and conventional LGE images (Table 2).

**Table 2:** Subjective assessment of presence of scar in the human subjects (Groups 1 and 2) and in the animals

	LV scar^a^			Papillary muscle scar^a^		
	GB-LGE	BB-LGE	Conventional LGE^b^	GB-LGE	BB-LGE	Conventional LGE^b^
Reader 1						
Group 1	9/18/0	9/17/1	6/19/2	14/13/0	12/15/0	10/17/0
Group 2	8/10/0	n.a.	6/12/0	3/15/0	n.a.	3/15/0
Animals	5/0/0	5/0/0	5/0/0	0/5/0	0/5/0	0/5/0
Reader 2						
Group 1	9/18/0	9/17/1	6/17/4	17/10/0	13/13/1	13/14/0
Group 2	8/10/0	n.a.	7/11/0	3/15/0	n.a.	3/15/0
Animals	5/0/0	5/0/0	5/0/0	0/5/0	0/5/0	0/5/0

^a^Data represent the number of cases scored as yes/no/uncertain regarding the presence of scar. Lack of BB-LGE data in group 2 is represented by ‘n.a.’ (i.e, not-available). *GB* gray blood, *BB* black blood, *LGE* late gadolinium enhancement

^b^LGE (humans) or phase-sensitive LGE (animals)

There was a statistically significant difference by the Friedman test between the subjective diagnostic value scores (i.e., the ability to detect LV scar, localize LV scar and detect papillary scar) among the three different sequences (Table 3 and Additional file 4: Table S2). Comparisons between each pair of sequences indicated that all subjective scores for the diagnostic value of GB-LGE were significantly higher than those of the conventional LGE images (*P* < 0.001 for all score comparisons). The GB-LGE scores were also significantly higher than those of BB-LGE images to localize LV scar (*P* = 0.024) and papillary muscle scar (*P* = 0.014). The GB-LGE was similar to BB-LGE in the ability to detect the LV scar (*P* = 0.10).

**Table 3:** Mean ± standard deviation of the subjective scores by two independent readers for different sequences and different criteria (i.e. LV scar detection, LV scar localization and papillary muscle scar detection) in the human dataset

	LV scar detection	LV scar localization	Papillary muscle scar detection
GB-LGE Score^a^	3.58 ± 0.74	3.67 ± 0.60	3.74 ± 0.61
BB-LGE Score^a^	3.59 ± 0.69	3.52 ± 0.70	3.59 ± 0.66
Conventional LGE Score^a^	2.99 ± 1.02	3.02 ± 0.97	3.08 ± 1.00
Friedman Test (*p*-value)	< 0.001	< 0.001	< 0.001
BB-LGE vs. LGE^b^	0.008	0.004	0.003
GB-LGE vs. LGE^b^	< 0.001	< 0.001	< 0.001
GB-LGE vs. BB-LGE^b^	0.1	0.024	0.014

^a^Data (mean ± standard deviation) are computed over the datasets using the mean score of the two readers. Score values are: 1 = Challenging, 2 = Difficult, 3 = Moderate, 4 = Easy. *GB* gray blood, *BB* black blood, *LGE* late gadolinium enhancement

^b^Data represent the p-value of the post hoc pairwise Wilcoxon signed-rank test

**Fig. 5:**
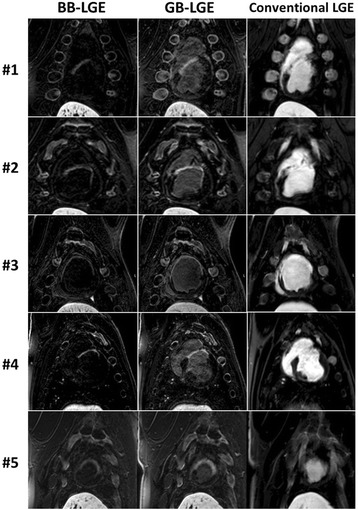
Comparison of black-blood (BB) late gadolinium enhancement (BB-LGE), gray-blood LGE (GB-LGE), and conventional (phase-sensitive inversion recovery) LGE images in the five swine with myocardial infarction. The scar location and extent can be best visualized in GB-LGE compared to the other two sequences

3D GB-LGE and 3D BB-LGE were successfully acquired in all animals (Fig. 5). The average scar-to-blood relative contrast in GB-LGE (1.5 ± 0.2) was higher than that of the conventional LGE images (− 0.03 ± 0.2) but lower than that in BB-LGE (5.2 ± 1.3) due to the complete nulling of the blood signal (Table 4). The scar-to-myocardium relative contrast in GB-LGE (8.9 ± 4.9) and conventional LGE (7.1 ± 6.2) were comparable but higher than that of BB-LGE (4.7 ± 1.5) (Table 4). The blood-to-myocardium relative contrast was reduced in GB-LGE (2.9 ± 1.7) and BB-LGE (0.1 ± 0.2) compared to conventional LGE (7.2 ± 5.9) (Table 4). Both reviewers assessed all images of diagnostic quality in all animals. There was no difference between readers in identifying hyper-enhancements in GB-LGE and BB-LGE images (Table 2). The subjective assessment of the ability to localize LV scar indicated that GB-LGE had consistently higher scores (4.0 ± 0.0) compared to BB-LGE (3.2 ± 0.5) and conventional LGE (2.0 ± 0.7) (Table 5).

**Table 4:** Scar-to-blood, scar-to-myocardium and blood-to-myocardium relative contrast in each animal dataset (in gray blood (GB), black blood (BB), and conventional (phase-sensitive inversion recovery) late gadolinium enhancement (LGE) sequences)

		Pig 1	Pig 2	Pig 3	Pig 4	Pig 5	Mean ± SD
Scar-to-blood	GB-LGE	1.76	1.27	1.56	1.33	1.73	1.53 ± 0.22
	BB-LGE	4.67	4.43	5.71	7.23	3.85	5.18 ± 1.33
	LGE	0.013	−0.19	−0.025	−0.17	0.21	−0.03 ± 0.16
Scar-to-myocardium	GB-LGE	17.59	4.80	6.91	7.66	7.87	8.97 ± 4.97
	BB-LGE	5.72	4.77	4.18	6.18	2.44	4.66 ± 1.47
	LGE	2.43	3.32	16.73	3.14	10.04	7.13 ± 6.19
Blood-to-myocardium	GB-LGE	5.73	1.55	2.09	2.72	2.25	2.87 ± 1.66
	BB-LGE	0.185	0.06	−0.23	−0.13	−0.29	0.08 ± 0.19
	LGE	2.38	4.3	17.19	3.99	8.12	7.20 ± 5.97

**Table 5:** Subjective evaluation scores of the ability to localize the scar in the each animal dataset in the gray blood (GB), black blood (BB), and conventional (phase-sensitive inversion recovery) late gadolinium enhancement (LGE) sequences

	GB-LGE^a^	BB-LGE^a^	Conventional LGE^a^
PIG 1	4	3	1
PIG 2	4	3	3
PIG 3	4	4	2
PIG 4	4	3	2
PIG 5	4	3	2
Mean ± SD	4.0 ± 0.0	3.2 ± 0.5	2.0 ± 0.7

^**a**^Data indicate the mean score of the two readers (the two readers’ scores were identical). Score values are: 1 = Challenging, 2 = Difficult, 3 = Moderate, 4 = Easy

## Discussion

We present a 3D GB-LGE sequence that yields improved scar visualization by increasing the contrast between scar, blood and normal myocardium. Phantom and in-vivo images show partial suppression of the blood signal can be achieved by choosing the appropriate sequence parameters. In our human datasets, GB-LGE contrast improved localization of the myocardium hyper-enhancements compared to BB-LGE and conventional LGE. In the animal datasets, despite the higher scar contrast in BB-LGE images, both readers consistently scored the GB-LGE images higher for the ability to localize LV scar compared to both BB-LGE and conventional LGE. While GB-LGE shares recently presented DB-LGE techniques the use of T_2_-preparation to adjust the image contrast [13, 15], it allows more flexibility to adjust the blood contrast compared to the method by Basha et al. [13].

Simple imaging parameter scouting was used in GB-LGE that included single breath-hold T_1_ mapping scan (9 heartbeats) and fast numerical solution of the optimization cost function (< 1 s). Compared to conventional LGE, GB-LGE requires additional steps of manual drawing of regions of interest to determine tissue T_1_ and running the optimization solver. This process usually takes 10-15 s and is performed by imaging technologists. The same concept of using T_1_ mapping for scouting the imaging parameters has been employed earlier in DB-LGE by Kellman et al. [15], where parameter computations were performed on the scanner main computer.

Both GB-LGE and BB-LGE data were acquired in 1 R-R (i.e. one heart-beat), while conventional PSIR-LGE images were acquired in 2 R-R. This resulted in improved signal-to-noise ratio (SNR) and fewer artifacts due to incorrect nulling. The 3D acquisition in GB-LGE and BB-LGE inherently improves the SNR despite the effect of T_2_ preparation pulses. However, 3D PSIR images will be 2× longer which will be clinically prohibitive especially for higher-spatial resolution scar imaging where scan time is 4-7 min [25–27]. That said, GB-LGE sequence can be easily modified to achieve PSIR GB-LGE, similar to BB-LGE [15]. Optimizing the contrast cost function in the proposed method results in shorter T_2_ preparation times (4-28 ms) compared to our prior parameter selection algorithm for DB-LGE (35 ms) [13] or PSIR-based DB-LGE (10-40 ms) [15]. A shorter T_2_ preparation time will result in increased SNR in the new parameters selection scheme; however we did not directly compare the SNR improvements between the two sequences.

In general, flow-independent DB-LGE imaging sequences can potentially generate GB-LGE contrast through some modifications. For example, increasing the time delay between T_2_ preparation and acquisition in DB-LGE [13] or changing the parameter selection function in PSIR DB-LGE [15] can result in GB-LGE contrast. However, this may require relaxing the constraints of fixing or minimizing the T_2_ preparation echo times in DB-LGE and PSIR DB-LGE, respectively. In other T_2_-prepared LGE imaging sequences [10–12], our parameter optimization methodology can be followed to generate the GB-LGE contrast. In PSIR DB-LGE (without magnetization preparation) method [9], increasing the inversion-to-acquisition time delay can generate GB-LGE contrast but will reduce the scar-to-blood relative contrast. In flow-based DB-LGE methods [6, 7], changing the sequence timing parameters results in incomplete blood suppression, which may lead to inhomogeneous and patchy signal within the blood pool.

In our study, we used different contrast agents, types and doses in animals and humans. Despite these differences, we successfully achieved GB-LGE in both human and animal studies. Use of a high-relaxivity contrast agent and dose in animal experiments resulted in higher blood pool signal in conventional PSIR-LGE images, and further demonstrated the effectiveness of GB-LGE in blood suppression and scar detection. This difference also highlights the robustness of the parameter optimization for parameter selection for different contrast types and doses. The proposed contrast function was intended to provide a simplified analytical approximation of the LGE image contrast to enable tractable optimization of the imaging parameters. The observed differences between the actual and the prescribed blood to scar contrast in GB-LGE in our study can be explained by several factors. First, the optimization solver was weighted to prioritize nulling the myocardium signal than achieving specific blood to scar ratio. Also, the actual scar T_1_ is controlled by many factors (including the dose and washout rate of the contrast material) while it is assumed fixed relative to the blood T_1_ in the contrast model. The latter factor leads to variability of the blood signal between patients. However, this effect also presents in conventional LGE but with a lesser extent (Additional files 1, 2, 3 and 4). This observation was confirmed in the quantified image contrast, where the standard deviation of the scar-to-blood relative contrast in GB-LGE was higher compared to conventional LGE (Table 1).

Our study has several limitations. While we found differences in the ability of different sequences to detect scarring, we do not have any histological evidence of the presence/extent of scarring in those patients. We have not performed histological validation of the GB-LGE sequence. Larger studies in patients are warranted to further assess the diagnostic and prognostic value of GB-LGE for scar detection.

## Conclusion

GB-LGE imaging improves the ability to identify and localize scarred tissues compared to BB-LGE and conventional LGE.

### Additional files

Additional file 1: The impact of fixing the cost function parameters on the optimal solution. (DOCX 36 kb)

Additional file 2: **Figure S1.** Comparison of the gray blood (GB) late gadolinium enhancement (GB-LGE), and conventional LGE images in four patients (axial views). Arrows indicate visible hyper-enhancements of right atrium (red), left ventricle (yellow), and chordae tendineae and papillary muscles (blue). (DOCX 739 kb)

Additional file 3: **Figure S2**. Comparison of gray blood (GB) late gadolinium enhancement (GB-LGE) acquired with two values of β (=0.05, and 0.1) and the conventional LGE images in four patients (axial views). Reducing the value of β results in darker blood signal intensity. (DOCX 1068 kb)

Additional file 4: **Table S2.** Mean ± SD of the subjective scores of the human datasets for each reader. (DOCX 15 kb)

## Appendix

In this work, a multi-shot spoiled gradient recalled echo acquisition sequence is employed with a train of *n* excitation RF pulses with a repetition time, *TR*; i.e. acquisition window= *n*. *TR*. Using Eq. 1, the signal intensity, ${S}_{acq^{-}}$, immediately before data acquisition is given by,

4 $$ {S}_{ac{q}^{-}}=1-{e}^{-\frac{D_3}{T_1}}+{e}^{-\frac{D_2}{T_2}}{e}^{-\frac{D_3}{T_1}}-\left(1+{M}_{ss}\right){e}^{-\frac{D_2}{T_2}}{e}^{-\frac{\left({D}_1+{D}_3\right)}{T_1}}. $$

In the above equation, the tissue type subscript, *r*, is dropped to simplify the mathematical notations. The longitudinal magnetization immediately before the *j* ^th^ RF pulse, ${S}_{j^{-}}$, is related to that before the (*j* − 1)^th^ RF pulse, ${S}_{{\left(j-1\right)}^{-}}$, by the following equation,

5 $$ {S}_{j^{-}}={S}_{{\left(j-1\right)}^{-}}\cos \left(\alpha \right){e}^{-\frac{TR}{T_1}}+\left(1-{e}^{-\frac{TR}{T_1}}\right), $$

where *α* is the flip angle, *j* =1, 2, … *n*, and ${S}_{0^{-}}={S}_{acq^{-}}$. After a sequence of *n* pulses, the longitudinal magnetization immediately after the *n* ^th^ pulse, ${S}_{acq^{+}}$, can be shown to be given by,

6 $$ {S}_{ac{q}^{+}}={S}_{ac{q}^{-}}{\left(\cos \left(\alpha \right){e}^{-\frac{TR}{T_1}}\right)}^n+\frac{1-{\left(\cos \left(\alpha \right){e}^{-\frac{TR}{T_1}}\right)}^n}{1-\cos \left(\alpha \right){e}^{-\frac{TR}{T_1}}}\left(1-{e}^{-\frac{TR}{T_1}}\right). $$

Due to the *T*_1_-relaxation during the time interval, *RX*, between the end of acquisition and the next inversion recovery pulse, the magnetization available for inversion is given by,

7 $$ {S}_{in{v}^{-}}={S}_{ac{q}^{+}}{e}^{-\frac{RX}{T_1}}+\left(1-{e}^{-\frac{RX}{T_1}}\right), $$

where, *RX* = *RR* − (*D*_1_ + *D*_2_ + *D*_3_) − *n*. *TR*, *RR* is the cardiac cycle duration, and ${S}_{inv^{-}}={M}_{ss}$ at steady state. Combining Eqs. 4, 6, and 7 leads to the following formula for *M*_*ss*_, which represents the magnetization available immediately before the inversion-recovery pulse,

8 $$ {M}_{ss}=\frac{1-{e}^{- RX/{T}_1}+A\left(1-{e}^{- TR/{T}_1}\right){e}^{- RX/{T}_1}+B\cos {\left(\alpha \right)}^n{e}^{\left( RR-{D}_1-{D}_2-{D}_3\right)/{T}_1}}{1+\cos {\left(\alpha \right)}^n{e}^{-{D}_2/{T}_2}{e}^{\left( RR-{D}_2\right)/{T}_1}}, $$

where,

9 $$ A=\frac{1-\cos {\left(\alpha \right)}^n{e}^{- nTR/{T}_1}}{1-\cos \left(\alpha \right){e}^{- TR/{T}_1}}, $$

10 $$ B=1-{e}^{-{D}_3/{T}_1}+{e}^{-{D}_2/{T}_2}{e}^{-{D}_3/{T}_1}-{e}^{-{D}_2/{T}_2}{e}^{-\left({D}_1+{D}_3\right)/{T}_1}. $$

## Abbreviations

3D: Three-dimensional
BB: Black blood
bSSFP: Balanced steady-state free precession
CMR: Cardiovascular magnetic resonance
DB: Dark blood
ECG: Electrocardiogram
FOV: Field of view
GB: Gray blood
GRE: Gradient recalled echo
HIPAA: Health insurance portability and accountability act
LAD: Left anterior descending coronary artery
LGE: Late gadolinium enhancement
LV: Left ventricle/left ventricular
MLEV: Malcom-Levitt
MOLLI: Modified Lock-Looker
PSIR: Phase sensitive inversion recovery
RF: Radiofrequency
SNR: Signal to noise ratio
TE: Echo time
TR: Repetition time

## References

[1] Kim RJ, Fieno DS, Parrish TB, Harris K, Chen EL, Simonetti O, Bundy J, Finn JP, Klocke FJ, Judd RM (1999). Relationship of MRI delayed contrast enhancement to irreversible injury, infarct age, and contractile function. Circulation.

[2] Han Y, Peters DC, Kissinger KV, Goddu B, Yeon SB, Manning WJ, Nezafat R (2010). Evaluation of papillary muscle function using cardiovascular magnetic resonance imaging in mitral valve prolapse. Am J Cardiol.

[3] Tanimoto T, Imanishi T, Kitabata H, Nakamura N, Kimura K, Yamano T, Ishibashi K, Komukai K, Ino Y, Takarada S (2010). Prevalence and clinical significance of papillary muscle infarction detected by late gadolinium-enhanced magnetic resonance imaging in patients with ST-segment elevation myocardial infarction. Circulation.

[4] Peters DC, Wylie JV, Hauser TH, Nezafat R, Han Y, Woo JJ, Taclas J, Kissinger KV, Goddu B, Josephson ME (2009). Recurrence of atrial fibrillation correlates with the extent of post-procedural late gadolinium enhancement: a pilot study. JACC Cardiovasc Imaging.

[5] Look DC, Locker DR (1970). Time saving in measurement of NMR and EPR relaxation times. Rev Sci Instrum.

[6] Farrelly C, Rehwald W, Salerno M, Davarpanah A, Keeling AN, Jacobson JT (2011). Improved detection of subendocardial hyperenhancement in myocardial infarction using dark blood-pool delayed enhancement MRI. AJR Am J Roentgenol.

[7] Yarnykh VL, Yuan C (2002). T1-insensitive flow suppression using quadruple inversion-recovery. Magn Reson Med.

[8] Peel SA, Morton G, Chiribiri A, Schuster A, Nagel E, Botnar RM (2012). Dual inversion-recovery MR imaging sequence for reduced blood signal on late gadolinium-enhanced images of myocardial scar. Radiology.

[9] Holtackers RJ, Chiribiri A, Schneider T, Higgins DM, Botnar RM (2017). Dark-blood late gadolinium enhancement without additional magnetization preparation. J Cardiovasc Magn Reson.

[10] Liu C-Y, Wieben O, Brittain JH, Reeder SB (2008). Improved delayed enhanced myocardial imaging with T2-prep inversion recovery magnetization preparation. J Magn Reson Imaging.

[11] Muscogiuri G, Rehwald WG, Schoepf UJ, Suranyi P, Litwin SE, De Cecco CN, Wichmann JL, Mangold S, Caruso D, Fuller SR (2017). T (Rho) and magnetization transfer and INvErsion recovery (TRAMINER)-prepared imaging: a novel contrast-enhanced flow-independent dark-blood technique for the evaluation of myocardial late gadolinium enhancement in patients with myocardial infarction. J Magn Reson Imaging.

[12] Kim HW, Rehwald WG, Wendell DC, Jenista E, Van Assche L, Jensen CJ (2016). Flow-Independent Dark-blood DeLayed Enhancement (FIDDLE): validation of a novel black blood technique for the diagnosis of myocardial infarction. J Cardiovasc Magn Reson.

[13] Basha TA, Tang MC, Tsao C, Tschabrunn CM, Anter E, Manning WJ, Nezafat R (2018). Improved dark blood late gadolinium enhancement (DB-LGE) imaging using an optimized joint inversion preparation and T2 magnetization preparation. Magn Reson Med.

[14] Basha T, Roujol S, Kissinger KV, Goddu B, Manning WJ, Nezafat R (2015). Black blood late gadolinium enhancement using combined T2 magnetization preparation and inversion recovery. J Cardiovasc Magn Reson.

[15] Kellman P, Xue H, Olivieri LJ, Cross RR, Grant EK, Fontana M, Ugander M, Moon JC, Hansen MS (2016). Dark blood late enhancement imaging. J Cardiovasc Magn Reson.

[16] Francis R, Kellman P, Kotecha T, Baggiano A, Norrington K, Martinez-Naharro A, Nordin S, Knight DS, Rakhit RD, Lockie T, Hawkins PN, Moon JC, Hausenloy DJ, Xue H, Hansen MS, Fontana M (2017). Prospective comparison of novel dark blood late gadolinium enhancement with conventional bright blood imaging for the detection of scar. J Cardiovasc Magn Reson.

[17] Kim HW, Rehwald WG, Jenista ER, Wendell DC, Filev P, Assche L, Jensen CJ, Parker MA, Chen E, Crowley ALC, Klem I, Judd RM, Kim RJ. Dark blood delayed-enhancement MRI of myocardial infarction. JACC Cardiovasc Imaging. 2017; 10.1016/j.jcmg.2017.09.021. 10.1016/j.jcmg.2017.09.021 PMC5993564 29248655

[18] Giri S, Chung Y-C, Merchant A, Mihai G, Rajagopalan S, Raman SV, Simonetti OP (2009). T2 quantification for improved detection of myocardial edema. J Cardiovasc Magn Reson.

[19] Wright GA, Hu BS, Macovski A (1991). Estimating oxygen saturation of blood in vivo with MR imaging at 1.5 T. J Magn Reson Imaging.

[20] Nocedal J, Wright SJ (2006). Numerical Optimization.

[21] Messroghli DR, Radjenovic A, Kozerke S, Higgins DM, Sivananthan MU, Ridgway JP (2004). Modified look-locker inversion recovery (MOLLI) for high-resolution T1 mapping of the heart. Magn Reson Med.

[22] Levitt MH, Freeman R, Frenkiel T (1982). Broadband heteronuclear decoupling. J Magn Reson.

[23] Moghari MH, Chan RH, Hong-Zohlman SN, Shaw JL, Goepfert LA, Kissinger KV, Goddu B, Josephson ME, Manning WJ, Nezafat R (2012). Free-breathing cardiac MR with a fixed navigator efficiency using adaptive gating window size. Magn Reson Med.

[24] Kellman P, Arai AE, Mcveigh ER, Aletras AH (2002). Phase-sensitive inversion recovery for detecting myocardial infarction using gadolinium-delayed hyperenhancement. Magn Reson Med.

[25] Akçakaya M, Basha TA, Goddu B, Goepfert LA, Kissinger KV, Tarokh V, Manning WJ, Nezafat R (2011). Low-dimensional-structure self-learning and thresholding: regularization beyond compressed sensing for MRI reconstruction. Magn Reson Med.

[26] Akçakaya M, Rayatzadeh H, Basha TA, Hong SN, Chan RH, Kissinger KV, Hauser TH, Josephson ME, Manning WJ, Nezafat R (2012). Accelerated late gadolinium enhancement cardiac MR imaging with isotropic spatial resolution using compressed sensing: initial experience. Radiology.

[27] Basha TA, Akçakaya M, Liew C, Tsao CW, Delling FN, Addae G, Ngo L, Manning WJ, Nezafat R (2017). Clinical performance of high-resolution late gadolinium enhancement imaging with compressed sensing. J Magn Reson Imaging.

