# Supplementary material for: Gray blood late gadolinium enhancement cardiovascular magnetic resonance for improved detection of myocardial scar
Source: J Cardiovasc Magn Reson. 2018 Mar 22;20:22. doi: 10.1186/s12968-018-0442-2 (PMC5863465; doi:10.1186/s12968-018-0442-2)
Supplement: Supplementary file 1 — The impact of fixing the cost function parameters on the optimal solution. (DOCX 36 kb) [file 12968_2018_442_MOESM1_ESM.docx]

**Effect of fixing scar T_1_ relative to the blood**

A numerical simulation was performed to study the blood-to-scar contrast variations associated with deviation of the actual scar T_1_ value, $T_{1\_act}^{scar}$ from that of the fixed values used to estimate the imaging parameters (i.e, $T_{1}^{scar}$ = 0.7 × $T_{1}^{blood}$). Similar to the simulation described in the main text, 10000 different combinations of tissues were generated by randomly selecting (using uniform probability distribution) the tissue T_1_ values from different continuous ranges for the blood ($T_{1}^{blood}$= 250-500 ms), myocardium ($T_{1}^{myo}$= 300-600 ms) and scar ($T_{1}^{scar}$= 250-500 ms). Only values satisfying $T_{1}^{r}<T_{1}^{myo}-100$ (with *r* = blood or scar) were used. Other simulation parameters included cardiac cycle duration= 1000 ms, flip angle = 25^o^, TR = 5 ms, number of RF pulses = 24 per cardiac cycle.

***GB-LGE simulation:*** The optimal timing parameters; i.e., *D_1_*, *D_2_*, and *D_3_*, are estimated using Equation (2) for each of the 10000 tissue combinations described above assuming T_2_= 50, 200 and 55 ms, for the myocardium, blood and scar (assuming $T_{1}^{scar}$ = 0.7 × $T_{1}^{blood}$), respectively. For simplicity the steady state magnetization was set to 1. The optimal parameters were then used to simulate the blood signal, *S_b_*, and scar signal, *S_s_*, using Equation (1). The scar signal was simulated at different actual scar T_1_ values, $T_{1\_act}^{scar}$, with$T_{1\_act}^{scar}/T_{1}^{blood}$= 0.5-0.85 with a step of 0.05. The (mean±standard deviation) of the ratio *S_b_/S_s_* was then calculated, over all tissue combinations, for each $T_{1\_act}^{scar}/T_{1}^{blood}$ ratio.

***Conventional LGE simulation:*** The effect of $T_{1\_act}^{scar}/T_{1}^{blood}$ on *S_b_/S_s_* in conventional LGE was simulated for comparison with that of GB-LGE. First, the inversion time was calculated as $TI=T_{1}^{myo}\times log(2)$ for each of the simulated 10000 tissue combinations. Then, the ratio *S_b_/S_s_* was calculated, where the scar signal was calculated at the different $T_{1\_act}^{scar}/T_{1}^{blood}$ ratios. The mean± standard deviation of the *S_b_/S_s_* ratio over the different tissue combinations was then calculated.

Figure S1 (below) shows a plot of *S_b_/S_s_* versus $T_{1\_act}^{scar}/T_{1}^{blood}$. The *S_b_/S_s_* values in the figure were normalized by the anticipated signal ratio (i.e., at $T_{1\_act}^{\mathrm{scar}}/T_{1}^{\mathrm{blood}}=0.7$). It can be noticed that variable $T_{1\_act}^{scar}/T_{1}^{blood}$ can result in variability in *S_b_/S_s_* in GB-LGE (0.54-1.73) and in conventional LGE (0.68-1.45).

In conclusion, fixing the ratio $T_{1}^{\mathrm{scar}}/T_{1}^{\mathrm{blood}}$ in the signal model of GB-LGE results in a deviation of the blood-to-scar contrast from the prescribed one. However, such contrast variability is inherent to LGE sequences due to the heavily T_1_-weighted contrast mechanism in these sequences.

Figure S1. A plot of the simulated blood-to-scar signal ratio (*S_b_/S_s_*) in gray blood (GB-) LGE and conventional LGE at different ratios of scar-to-blood T_1_ values ($T_{1\_act}^{\mathrm{scar}}/T_{1}^{\mathrm{blood}}$). Data points and error bars respectively represent mean and standard deviation of S_b_/S_s_ calculated over 10000 different tissue combinations.

**Effect of fixing T_2_**

A numerical simulation was performed to study the tissue signal variations associated with deviation of the actual tissue T_2_ from that of the fixed values used to estimate the imaging parameters. First, a set of T_2_ values is randomly (using uniform probability distribution) selected for the blood, myocardium and scar from the continuous ranges 180-220 ms, 40-55 ms, and 40-60 ms, respectively. The optimal timing parameters; i.e., *D_1_*, *D_2_*, and *D_3_*, are estimated using Equation (2) for each of the 1000 tissue combinations described above assuming T_2_= 50, 200 and 55 ms, for the myocardium, blood and scar, respectively. Then, Equation (1) was used to simulate each tissue signal twice using: (1) the actual T_2_, and (2) the assumed T_2_ values. In both cases, a Rician model was used to generate a noisy tissue signal with signal-to-noise ratio of 5, defined as (signal mean) / (noise SD). The (mean±SD) of each tissue signal was calculated (Table S1). A two-tail paired t-test was used to compare T_2_-related signal variations. The results showed that the discrepancy among the assumed and the actual T_2_ values had no significant effect on the tissue contrast in both the BB-LGE and GB-LGE (Table S1).

**Table S1.** Signal intensity of each tissue type in GB-LGE and BB-LGE.

|  | **scar** | | | **blood** | | | **Myocardium** | | |
| --- | --- | --- | --- | --- | --- | --- | --- | --- | --- |
|  | **Actual T_2_** | **Assumed T_2_** | ***P*** | **Actual T_2_** | **Assumed T_2_** | ***P*** | **Actual T_2_** | **Assumed T_2_** | ***P*** |
| **GB-LGE** | 0.8 ±0.3 | 0.8 ±0.3 | 0.36 | 0.25 ±0.05 | 0.25 ±0.04 | 0.40 | 0.04 ±0.03 | 0.02 ±0.01 | 0.53 |
| **BB-LGE** | 0.8 ±0.3 | 0.8 ±0.3 | 0.63 | 0.08 ±0.04 | 0.08 ±0.03 | 0.54 | 0.05 ±0.04 | 0.02 ±0.01 | 0.67 |

Note—Values represent mean±SD of the tissue signal (arbitrary units) calculated using the actual T_2_ and the assumed T_2_. *P* values are for the hypothesis test of the equality of the two signal means (estimated using actual T_2_ vs. assumed T_2_).
