# Supplementary material for: Gray blood late gadolinium enhancement cardiovascular magnetic resonance for improved detection of myocardial scar
Source: J Cardiovasc Magn Reson. 2018 Mar 22;20:22. doi: 10.1186/s12968-018-0442-2 (PMC5863465; doi:10.1186/s12968-018-0442-2)
Supplement: Supplementary file 2 — Figure S1. Comparison of the gray blood (GB) late gadolinium enhancement (GB-LGE), and conventional LGE images in four patients (axial views). Arrows indicate visible hyper-enhancements of right atrium (red), left ventricle (yellow), and chordae tendineae and papillary muscles (blue). (DOCX 739 kb) [file 12968_2018_442_MOESM2_ESM.docx]

**
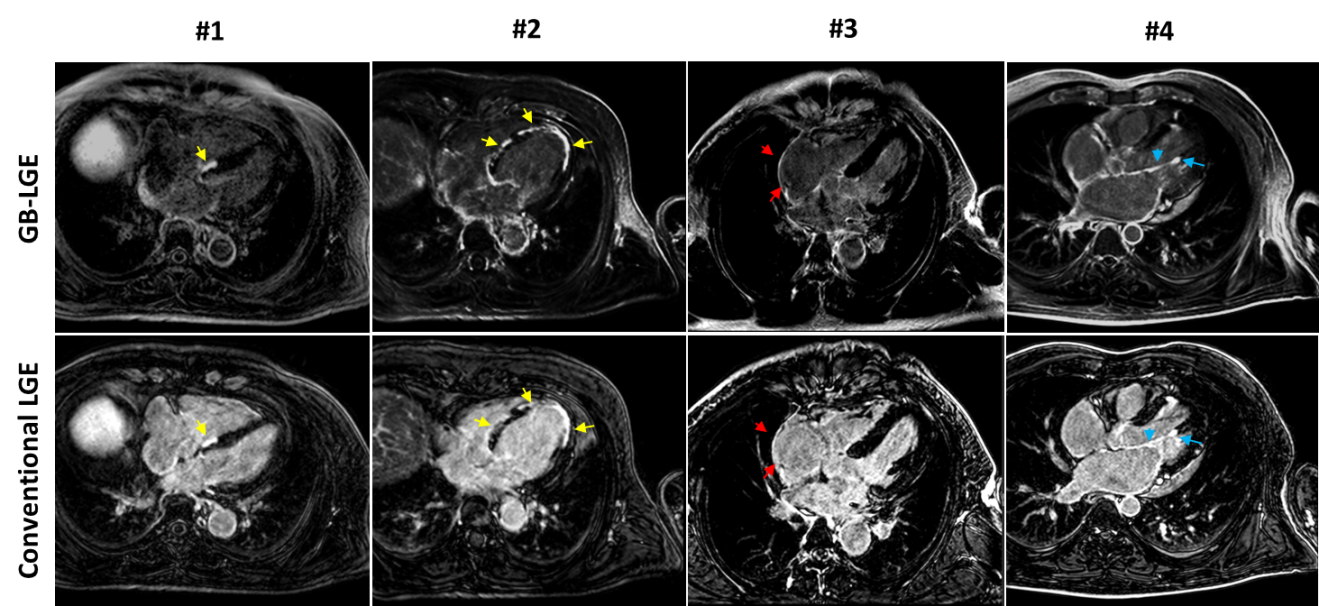
**

**Figure S1.** Comparison of the gray blood (GB) late gadolinium enhancement (GB-LGE), and conventional LGE images in four patients (axial views). Arrows indicate visible hyper-enhancements of right atrium (red), left ventricle (yellow), and chordae tendineae and papillary muscles (blue).
