# Supplementary material for: Gray blood late gadolinium enhancement cardiovascular magnetic resonance for improved detection of myocardial scar
Source: J Cardiovasc Magn Reson. 2018 Mar 22;20:22. doi: 10.1186/s12968-018-0442-2 (PMC5863465; doi:10.1186/s12968-018-0442-2)
Supplement: Supplementary file 3 — Figure S2. Comparison of gray blood (GB) late gadolinium enhancement (GB-LGE) acquired with two values of β (=0.05, and 0.1) and the conventional LGE images in four patients (axial views). Reducing the value of β results in darker blood signal intensity. (DOCX 1068 kb) [file 12968_2018_442_MOESM3_ESM.docx]

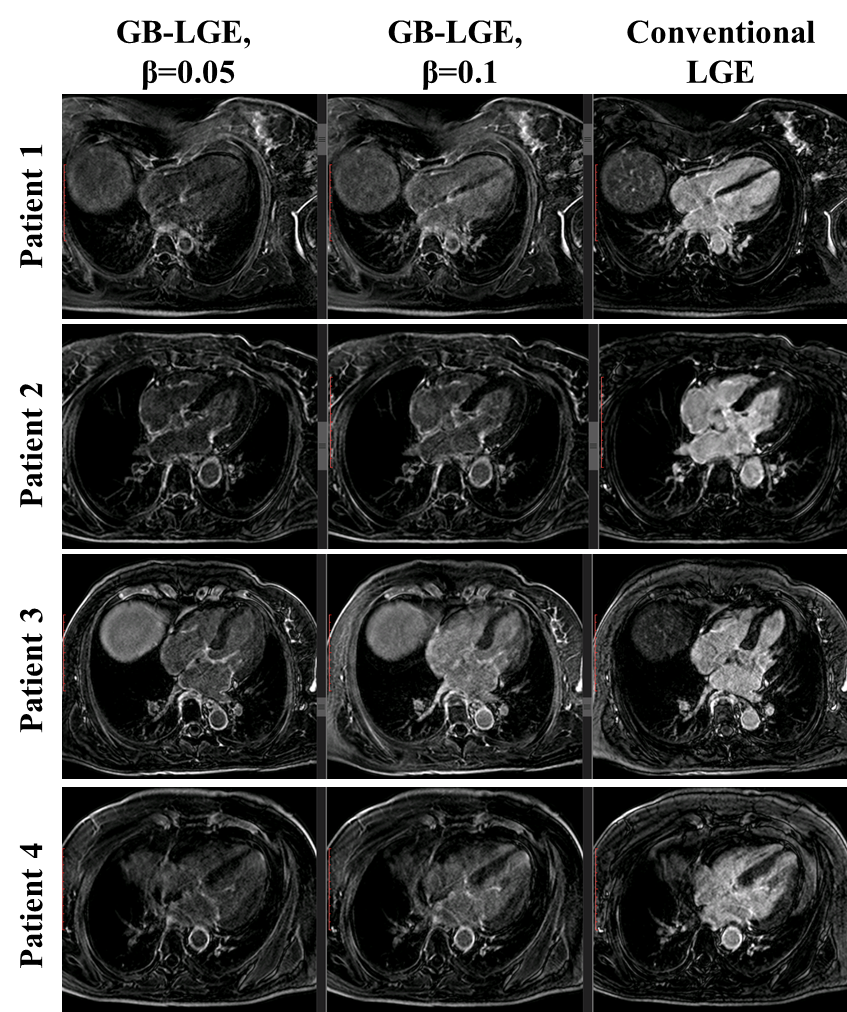


**Figure S2**. Comparison of gray blood (GB) late gadolinium enhancement (GB-LGE) acquired with two values of β (=0.05, and 0.1) and the conventional LGE images in four patients (axial views). Reducing the value of β results in darker blood signal intensity.
