# Supplementary material for: Gray blood late gadolinium enhancement cardiovascular magnetic resonance for improved detection of myocardial scar
Source: J Cardiovasc Magn Reson. 2018 Mar 22;20:22. doi: 10.1186/s12968-018-0442-2 (PMC5863465; doi:10.1186/s12968-018-0442-2)
Supplement: Supplementary file 4 — Table S2. Mean ± SD of the subjective scores of the human datasets for each reader. (DOCX 15 kb) [file 12968_2018_442_MOESM4_ESM.docx]

**Table S2.** Mean±SD of the subjective scores of the human datasets for each reader.

|  |  | Ability to detect LV scar^a^ | Ability to localize LV scar^a^ | | Ability to detect papillary scar^a^ | |
| --- | --- | --- | --- | --- | --- | --- |
| Reader 1 | **BB-LGE (n=27)** | 3.59±0.69 | | 3.59±0.69 | | 3.59±0.63 |
|  | **GB-LGE (n=45)** | 3.56±0.76 | | 3.56±0.76 | | 3.73±0.62 |
|  | **Conventional LGE (n=45)** | 2.98±1.03 | | 2.98±1.03 | | 3.11±0.91 |
| Reader 2 | **BB-LGE (n=27)** | 3.59±0.69 | | 3.56±0.64 | | 3.59±0.69 |
|  | **GB-LGE (n=45)** | 3.60±0.72 | | 3.67±0.60 | | 3.76±0.61 |
|  | **Conventional LGE (n=45)** | 3.00±1.02 | | 3.07±0.96 | | 3.04±1.09 |

a Data (average ± standard deviation) are computed over the different human datasets. Score values are: 1=Challenging, 2=Difficult, 3=Moderate, 4=Easy. GB= gray blood, BB=black blood, LGE= late gadolinium enhancement.
